# Bone marker gene expression in calvarial bones: different bone microenvironments

**DOI:** 10.1186/s40709-017-0066-y

**Published:** 2017-05-16

**Authors:** Osama Al-Amer

**Affiliations:** Department of Medical Laboratory Technology, Faculty of Applied Medical Sciences, University of Tabuk, Tabuk, Kingdom of Saudi Arabia

**Keywords:** Calvariae, Bone microenvironment, Gene markers, Gene expression

## Abstract

**Background:**

In calvarial mice, mesenchymal stem cells (MSCs) differentiate into osteoprogenitor cells and then differentiate into osteoblasts that differentiate into osteocytes, which become embedded within the bone matrix. In this case, the cells participating in bone formation include MSCs, osteoprogenitor cells, osteoblasts and osteocytes. The calvariae of C57BL/KaLwRijHsD mice consist of the following five bones: two frontal bones, two parietal bones and one interparietal bone. This study aimed to analyse some bone marker genes and bone related genes to determine whether these calvarial bones have different bone microenvironments.

**Methods:**

C57BL/KaLwRijHsD calvariae were carefully excised from five male mice that were 4–6 weeks of age. Frontal, parietal, and interparietal bones were dissected to determine the bone microenvironment in calvariae. Haematoxylin and eosin staining was used to determine the morphology of different calvarial bones under microscopy. TaqMan was used to analyse the relative expression of Runx2, OC, OSX, RANK, RANKL, OPG, N-cadherin, E-cadherin, FGF2 and FGFR1 genes in different parts of the calvariae.

**Results:**

Histological analysis demonstrated different bone marrow (BM) areas between the different parts of the calvariae. The data show that parietal bones have the smallest BM area compared to frontal and interparietal bones. TaqMan data show a significant increase in the expression level of Runx2, OC, OSX, RANKL, OPG, FGF2 and FGFR1 genes in the parietal bones compared with the frontal and interparietal bones of calvariae.

**Conclusion:**

This study provides evidence that different calvarial bones, frontal, parietal and interparietal, contain different bone microenvironments.

## Background

C57BL/KalwRijHsD mice develop a high frequency of monoclonal proliferative B cell disorders. Most C57BL/KalwRijHsD mice have a monoclonal gammopathy of undetermined significance (MGUS) that is similar to humans. The 5TMM series of myeloma models originate from spontaneously developed multiple myeloma (MM) in ageing C57BL/KalwRijHsD mice and have many of the features of the human disease [1–3]. Several murine models of MM exist, but 5T2, 5T33 and 5TGM1 are the best characterized and have been used in most recent studies [4–10]. Al-Amer (2015) recently used a confocal laser scanning microscope LSM 510, multiphoton microscopy, to trace the homing of DiD^+^ myeloma cells and determine the distribution of these cells in calvarial bone BM. The data showed myeloma cells arrive to all parts of calvariae after 3 days of injection [11]. However, in another study, the same author showed myeloma tumours do not grow in all parts of the calvariae [12]. These findings raised the question of whether parts of calvariae have different bone microenvironments.

Osteoblasts are mononucleate cells derived from putative MSCs that are responsible for bone formation and regulation of osteoclast differentiation. Bone morphogenic proteins (BMPs), Wnt/β-catenin and Notch signalling play a major role in osteoblastogenesis [13–15]. In addition, Runt-related transcription factor 2 (RUNX2) has been shown to be an essential transcription factor for osteoblastogenesis. It was found that Runx2−/− mice failed to develop calvarial bones, and the mice died at birth. Runx2 also regulates another transcription factor that is necessary for osteoblastogenesis, osterix (OSX) [16]. Mature osteoblasts are identified by their cuboidal structure and location on the endosteal surface. Type I collagen is the major product of the bone-forming osteoblast in addition to osteocalcin (OC) [17].

Bone remodelling is a complex process in which mature bone tissue is removed by a process called bone resorption, and new bone tissue is formed by a process called bone formation. In this process, cellular activity and molecular mechanisms are closely coordinated to ensure the bone resorption-formation sequence is performed at a mutual location, sustaining the bone mass. Osteoclastic bone resorption is controlled by receptor activator of nuclear factor kappa-B ligand (RANKL). The binding of RANKL to RANK starts the osteoclastogenesis process and stimulates osteoclast activity and bone resorption [18]. Osteoprotegerin (OPG), a soluble tumour necrosis factor (TNF) receptor superfamily, is produced by many cell types, such as bone-marrow stromal cells and osteoblasts. OPG binds to RANKL and protects the bone from excessive resorption by preventing the RANK/RANKL interaction [19–21]. It was determined that overexpression of OPG reduces osteoclastogenesis in mice; however, a lack of OPG has been shown to accelerate osteoclastogenesis, and OPG Ko mice (*OPG*−/− mice) develop severe osteoporosis [22].

Neural (N)-cadherin is an adhesion molecule that has been implicated in the localization of haematopoietic stem cells (HSCs) to ‘niches’ containing osteoblasts on endosteal bone surfaces. HSCs home into the endosteal surfaces and attach to N-cadherin^+^ osteoblasts. This attachment keeps the HSCs in a quiescent state in the HSC niche [23, 24]. Epithelial (E-) cadherin plays an essential role in Ca^2+^-dependent cell-to-cell adhesion. A striking feature of the type I (classic) cadherins is a highly conserved His-Ala-Val (HAV) motif in the first extracellular domain that mediates homophilic interactions between cadherin molecules expressed on adjacent cells [25]. On the other side of the cell membrane, the cadherin intracellular domain forms a protein complex with catenin molecules, which anchor the cadherins to the cytoskeleton and provide a unique signal transduction pathway for cadherin interactions [26].

Fibroblast growth factor receptors (FGFRs) play major roles in skeletogenesis, and activating mutations of the human FGFR1, FGFR2 and FGFR3 genes cause premature fusion of the skull bones (craniosynostosis) [27]. This genetic evidence establishes a role for FGFR1 in skeletal development and suggests that the FGFR1, FGFR2 and FGFR3 signalling pathways may have similar or redundant functions. In addition to premature fusion of cranial sutures, several of the classic craniosynostosis syndromes have associated phenotypes that affect long bone development in the appendicular skeleton. This genetic evidence establishes a role for FGFR1 in skeletal development and suggests that the FGFR1, FGFR2 and FGFR3 signalling pathways may have similar or redundant functions. In addition to premature fusion of cranial sutures, several of the classic craniosynostosis syndromes have associated phenotypes that affect long bone development in the appendicular skeleton [28]. FGFRs 1–3 are expressed in the developing and mature skeleton in patterns suggestive of both unique and redundant function [29].

In the present study, I evaluated the relative expression levels of osteoblast markers (Runx2, OC and OSX) and the regulation of bone resorption molecules (RANK, RANKL and OPG), adhesion molecules (N-cadherin and E-cadherin) and skeletogenesis markers (FGF2 and FGFR1) in different parts of the calvariae. The osteoblast markers (Runx2, OC, and OSX) and the regulation of bone resorption markers (RANKL and OPG) and skeletogenesis markers (FGF2 and FGFR1) are significantly highly expressed in parietal bone compared with frontal and interparietal bones. However, there were no differences in the relative expression levels of the adhesion molecules (N-cadherin and E-cadherin) between the frontal, parietal and interparietal bones. This study provides evidence that different calvarial bones contain different bone microenvironments.

## Methods

### Animals

Male C57BL/KalwRiJHsD mice, aged 4–6 weeks, were purchased from Harlan, Netherlands and from the University of Leeds, UK. Mice were housed in the University of Sheffield biological services laboratory. All animals were provided with food and water ad libitum as well as light, and all procedures were performed under a personal license (40/10118).

### Study plan

C57BL/KaLwRijHsd calvariae were carefully excised from five male mice that were 4–6 weeks old. Mice were handled carefully. The frontal, parietal, and interparietal bones were carefully dissected, and sections of each calvarial part were cut for three levels. Each level cut was 200 μm after the previous level. The total sample size was 45, including 15 samples from frontal bones, 15 from parietal bones and 15 from interparietal bones. Each sample was used in triplicate during TaqMan analysis.

### Haematoxylin and eosin staining

Haematoxylin and eosin stain (H&E) is a standard staining method in histological studies. Haemalum is formed from aluminium ions and oxidized haematoxylin stains nuclei with a blue colour, while eosin stains the cytoplasm and extracellular matrix with varying degrees of pink [30, 31]. Bone sections were dipped twice in xylene for 5 min to remove wax and rehydrated twice in absolute alcohol, 95% alcohol and 70% alcohol for 5 min before they were briefly washed in water. Gill’s II haematoxylin (VWR, UK) was used for 120 s to stain nuclei. Sections were washed in water. Then, 1% aqueous eosin (VWR, UK) with 1% calcium carbonate (Sigma, UK) was used for 5 min to stain the cytoplasm. Sections were dehydrated in 70% alcohol for 10 s, 95% alcohol for 10 s and twice in absolute alcohol for 30 s each. Xylene was used for 1 min to remove alcohol and for 3 min for clearance. Slides were mounted and covered using coverslips (VWR, UK). In the endo-cortical and trabecular surfaces of the calvariae, the nuclei were stained blue and the cytoplasm and extracellular matrices were stained pink.

### High-resolution micro-computed tomography (μCT)

A high resolution μCT scanner (model 1172; Skyscan, Belgium) is a non-destructive technique that provides three-dimensional bone microstructure. A μCT scanner was used to scan calvarial bones. Image were captured every 0.35° through a 360° rotation with a pixel size of 4.1. Scanned images were reconstructed using Skyscan NRecon software (version 1.5.1.3, Skyscan, Belgium).

### Leitz DMRB microscopy

Osteomeasure software version 4.10 (Osteomerics Incorporated, Atlanta, USA) was used with a Leitz DMRB microscope and a drawing tablet (CalComp Drawing Broad III) to quantify the BM area of calvariae. This microscopy was used to visualize non-fluorescent cells and sections.

### TaqMan analysis

Total RNA was isolated from primary osteoblasts at different stages of development using TRIzol. RNAs were transcribed to cDNA using SuperScript™ II reverse transcriptase (Invitrogen, UK). TaqMan probes for Runx2, OC, OSX, RANK, RANKL, OPG, N-cadherin, E-cadherin, FGF2 and FGFR1 were used to quantify different genes with TaqMan Universal Master Mix II (Applied Biosystems, UK) and normalized with the level of housekeeping gene, β-actin. An ABI PRISM >900 sequence detector was used to quantify the expression of Runx2, OC, OSX, RANK, RANKL, OPG, N-cadherin, E-cadherin, FGF2 and FGFR1 in different calvarial bones.

### Statistical analysis

Statistical significance was determined using Tukey’s test using GraphPad Prism 6 (GraphPad Software Inc., California, USA). Data were considered significant for *p* ≤ 0.05 (* *p* < 0.05, ** *p* < 0.01, and *** *p* < 0.001). All data were presented as the mean ± standard deviation (±SD).

## Results

### Huge BM area in interparietal bone compared with frontal and parietal bones

**Fig. 1:**
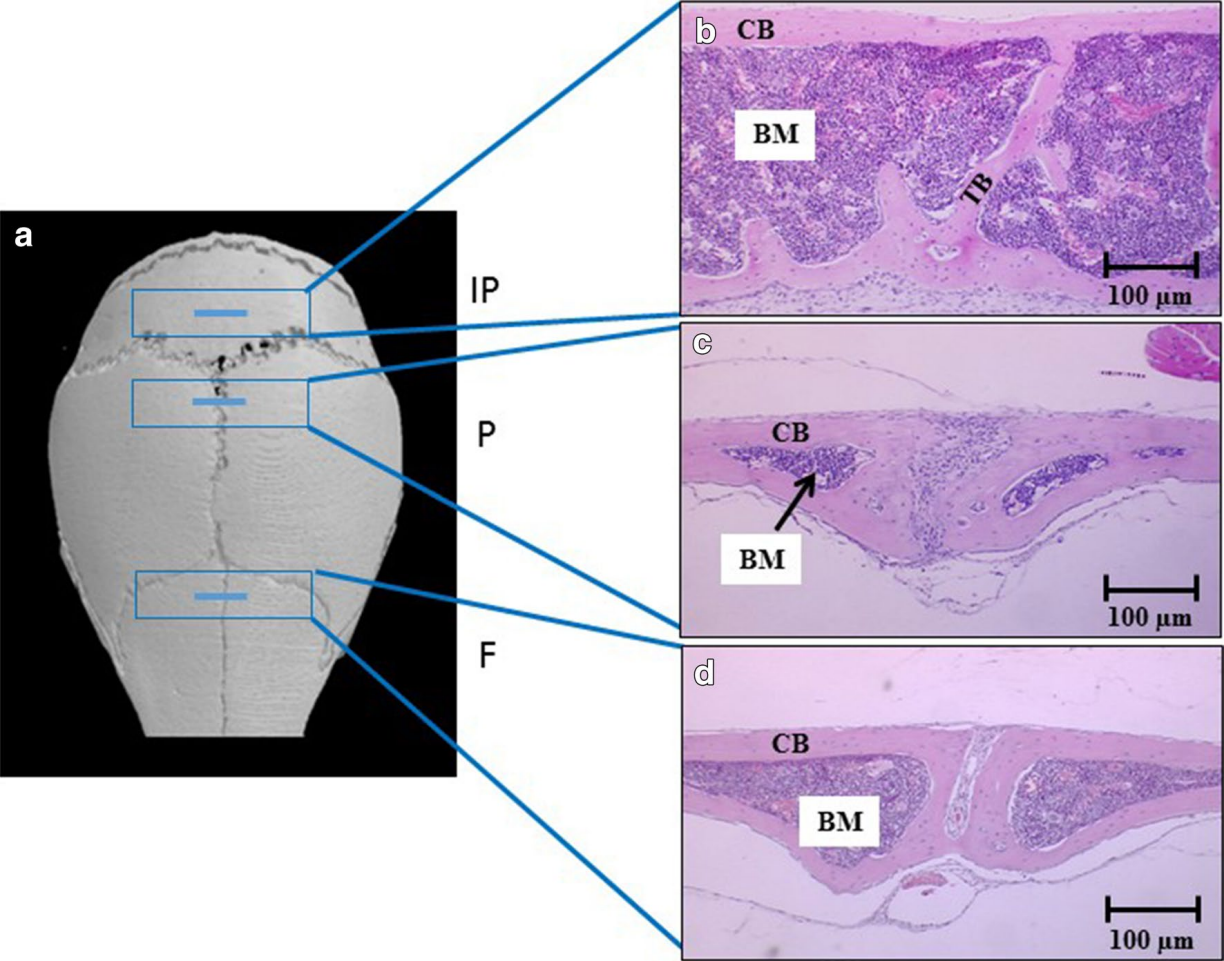
Sectioning of calvarial bones across the regions of interest. **a** MicroCT model of a murine skull showing calvariae consisting of frontal (F), parietal (P) and interparietal (IP) bones. **b**–**d** Cross-sections of frontal, parietal and interparietal bones, respectively. *BM* is bone marrow, *CB* is cortical bone and *TB* is trabecular bone

**Fig. 2:**
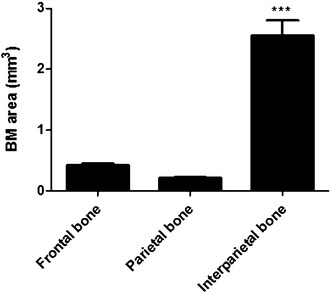
Analysis of BM areas in calvarial bones of male C57BL/KalwRiJHsD mice. There was a significant increase in the BM volume in interparietal bones compared to frontal and parietal bones

The BM area in C57BL/KaLwRijHsD calvariae was determined. Cortical bone was identified as an outside solid bone with a regular structure, while trabecular bone was identified as a bone inside the BM with an irregular structure (Fig. 1). The endo-cortical bone surface is a surface of the cortical bone inside the BM. Histological analysis demonstrated statistically significant increases in the BM area in interparietal bones compared to frontal and parietal bones (Fig. 2).

### Relative level expression of Runx2, OC and OSX in calvarial bones

**Fig. 3:**
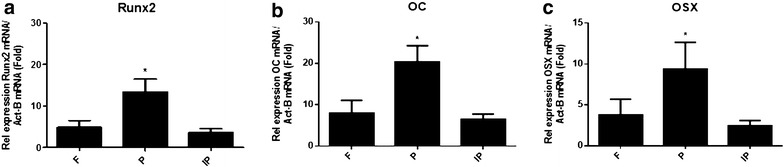
TaqMan analysis of osteoblast markers: Runx2, OC and OSX expression in calvariae of C57BL/KalwRiJHsD mice. RNA was extracted with cDNA synthesized from frontal (F), parietal (P) and interparietal (IP) bones. The relative expression of osteoblast marker genes was adjusted to the relative expression of a housekeeping gene, β-actin. Data showed that there was a significant increase in the relative expression levels of Runx2 (**a**), OC (**b**) and OSX (**c**) in parietal bone compared to frontal and interparietal bones

The expression levels of osteoblast markers, Runx2, OC and OSX, were analysed using TaqMan. Expression of these genes was normalized to the expression of a housekeeping gene, β-actin. Figure 3a shows a significant increase in the relative expression of Runx2 gene in the parietal bones compared with the frontal and interparietal bones. Figure 3b shows a significant increase in the relative expression of the OC gene in the parietal bones compared with the frontal and interparietal bones. Figure 3c shows a significant increase in the relative expression of the OSX gene in the parietal bones compared with the frontal and interparietal bones.

### Relative expression levels of RANK, RANKL and OPG in calvarial bones

**Fig. 4:**
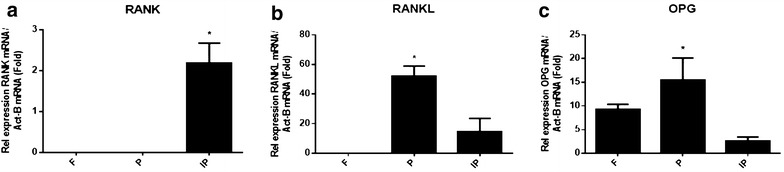
TaqMan analysis of bone resorption molecules: RANK, RANKL and OPG expression in calvariae of C57BL/KalwRiJHsD mice. RNA was extracted with cDNA synthesized from frontal (F), parietal (P) and interparietal (IP) bones. The relative expression of osteoclast marker genes was adjusted to the relative expression of a housekeeping gene, β-actin. Data show that the RANK gene was only expressed in interparietal bone, while it was not expressed in frontal or parietal bones. Additionally, there was a significant increase in the relative expression of the RANK gene (**a**) in interparietal bone compared to frontal and parietal bones. On the other hand, there was a significant increase in the relative expression of the RANKL (**b**) and OPG (**c**) genes in parietal bone compared to frontal and interparietal bones

The expression levels of bone resorption regulation molecules, RANK, RANKL and OPG, were analysed using TaqMan. The expression of these genes was normalized to the expression of a housekeeping gene, β-actin. Figure 4a shows that RANK was only expressed in interparietal bone, but it was not expressed in frontal or parietal bone. Figure 4b shows that RANKL was expressed in parietal and interparietal bones, but it was not expressed in frontal bone. Data show a significant increase in the relative expression of the RANKL gene in the parietal bones compared with the frontal and interparietal bones. Figure 4c shows a significant increase in the relative expression of the OPG gene in the parietal bones compared with the frontal and interparietal bones.

### Relative expression levels of N-cadherin and E-cadherin in calvarial bones

**Fig. 5:**
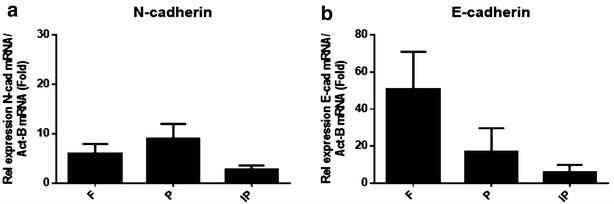
TaqMan analysis of adhesion molecules: N-cadherin and E-cadherin expression in calvariae of C57BL/KalwRiJHsD mice. RNA was extracted with cDNA synthesized from frontal (F), parietal (P) and interparietal (IP) bones. The relative expression of adhesion molecule was adjusted to the relative expression of a housekeeping gene, β-actin. There were no differences in the relative expression of N-cadherin (**a**) and E-cadherin (**b**) between frontal, parietal and interparietal bones

The expression levels of adhesion molecules, N-cadherin and E-cadherin, were analysed using TaqMan. The expression of these genes was normalized to the expression of a housekeeping gene, β-actin. Both genes are expressed in all parts of calvariae. Figure 5a shows a non significant increase in the relative expression of the N-cadherin gene in the parietal bones compared with the frontal and interparietal bones. Figure 5b shows an increase in the relative expression of the E-cadherin gene in the frontal bones compared with the parietal and interparietal bones, but it was not significant, although *p* value was close to significant (*p* = 0.06).

### Relative expression levels of FGF2 and FGFR1 in calvarial bones

**Fig. 6:**
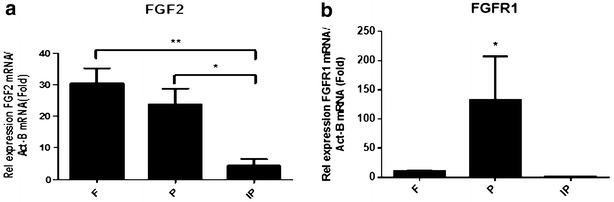
TaqMan analysis of skeletogenesis markers: FGF2 and FGFR1 expression in calvariae of C57BL/KalwRiJHsD mice. RNA was extracted with cDNA synthesized from frontal (F), parietal (P) and interparietal (IP) bones. The relative expression of skeletogenesis marker genes was adjusted to the relative expression of a housekeeping gene, β-actin. There was a significant increase in the relative expression of FGF2 (**a**) in frontal and parietal bones compared to interparietal bone. In addition, there was no difference in the relative expression of FGF2 between frontal and parietal bones. On the other hand, the relative expression of FGFR1 (**b**) genes was significantly expressed in parietal bone compared to frontal and interparietal bones

The expression levels of skeletogenesis markers, FGF2 and FGFR1, were analysed using TaqMan. The expression of these genes was normalized to the expression of a housekeeping gene, β-actin. Figure 6a shows a significant increase in the relative expression of the FGF2 gene in the frontal and parietal bones compared with the interparietal bones, but there was no significant different in the relative expression between the frontal and parietal bones. Figure 6b shows a significant increase in the relative expression of the FGFR1 gene in the parietal bones compared with the frontal and interparietal bones.

## Discussion

In this study, different calvariae—frontal, parietal and interparietal—were analysed. The aim of this study was to test the hypothesis that calvarial bones contain different bone microenvironments. In previous studies, C57BL/KaLwRijHsd mice were inoculated with myeloma cells via their tail vein. Multi-photon microscopy demonstrated a significant increase in the number of myeloma cells that homed to the interparietal bones 3 and 21 days post inoculation compared to frontal and parietal bones [11]. In a separate study, C57BL/KaLwRijHsd mice were also inoculated with myeloma cells via their tail veins. Using the Lightools image analysis system with ImageJ software demonstrated that a large tumour was formed in interparietal bones and not in frontal or parietal bones after 3 weeks [12].

To date, no publication has demonstrated whether different calvarial bones—frontal, parietal or interparietal—contain different bone microenvironments in C57BL/KaLwRijHsD mice. Parfitt et al. (1987) summarized a unified system of terminology for bone histomorphometry. The bone histomorphometry community decided to measure the BM volume as the marrow area (Ma.Ar) [32]. According to this terminology, the BM areas of the frontal, parietal and interparietal regions of calvariae were analysed in this study. It has been reported that calvariae of C57BL/KaLwRijHsD mice consist of the following five bones: two frontal bones, two parietal bones and one interparietal bone [11]. In this study, the BM area of different parts of C57BL/KaLwRijHsd calvariae was analysed under a microscope. Interestingly, data showed that there was a significant increase in the BM area of interparietal bones compared to frontal and parietal bones. Together, these studies suggest the possibility that there are different bone microenvironments in the calvariae.

Healthy bone is regulated and coordinated by a bone remodelling cycle consisting of bone formation and bone resorption throughout the skeleton. One of the most important consequences of cancer metastasis in the bone is the induced imbalance in bone remodelling between osteoblasts and osteoclasts, which affects bone resorption and bone formation. This imbalance between osteoclasts and osteoblasts is responsible for an increase in bone resorption and a reduction in bone formation, resulting in osteolytic bone lesions [33–36]. C57BL/KalwRij mice are a pre-clinical model used to study MM bone disease. Most C57BL/KalwRij mice have a monoclonal gammopathy of undetermined significance (MGUS) similar to humans [1–3]. To support our understanding of the bone microenvironment in the pre-clinical model to study MM bone disease, the relative expression levels of osteoblast markers (Runx2, OC and OSX) and the regulation of bone resorption molecules (RANK, RANKL and OPG), adhesion molecules (N-cadherin and E-cadherin) and skeletogenesis markers (FGF2 and FGFR1) in different calvariae were analysed.

It has been reported that Runx2, OC and OSX play an important role in osteoblastogenesis, and a deficiency in one of them plays a critical role in murine bone development [16, 17]. On the other hand, it has been reported that the RANKL/RANK/OPG system plays an important role in bone resorption. RANKL/RANK signalling promotes osteoclast differentiation and activation, leading to bone resorption. OPG binds to RANKL and protects bone from excessive resorption [18–22]. For osteoblast markers (Runx2, OC and OSX), this study demonstrated that there was a significant increase in the relative expression levels of Runx2, OC and OSX in parietal bones compared with frontal and interparietal bones. For bone resorption regulation molecules (RANK, RANKL and OPG), this study demonstrated that there was a significant increase in the relative expression levels of RANKL and OPG in parietal bones compared to frontal and interparietal bones. However, the expression of RANK was significantly higher in interparietal bones compared with frontal and parietal bones. These findings show that the parietal bone has a high expression level of osteoblast markers (Runx2, OC and OSX) and RANKL/OPG, blocking bone resorption. This could explain the finding that there is a small area of bone marrow in parietal bone that shows more bone formation and blocks bone resorption. On the other hand, interparietal bone has a low expression level of osteoblast markers (Runx2, OC and OSX) in addition to a high expression level of RANK. This could explain the finding that there is a large bone marrow area in interparietal bone where there is more bone resorption and less bone formation. This study also showed that there is a significant increase in skeletogenesis markers (FGF2 and FGFR1) in parietal bones. On the other hand, this study showed that there were no differences in the relative expression of the adhesion molecules (N-cadherin and E-cadherin) between the frontal, parietal and interparietal bones.

## Conclusion

This study provides evidence that different calvarial bones—frontal, parietal and interparietal—contain different bone microenvironments. This provides platforms for pre-clinical investigations to study myeloma colonization and growth in the skull (calvarial bone) as well as anti-myeloma therapies, particularly those targeting myeloma bone disease.
